# Hydrogels Powered by Nanoemulsion Technology for the Topical Delivery of *Acmella oleracea* Extract

**DOI:** 10.3390/pharmaceutics17050625

**Published:** 2025-05-08

**Authors:** Eleonora Spinozzi, Marco Cespi, Marta Ferrati, Riccardo Petrelli, Filippo Maggi, Junbiao Wang, Sunday Segun Alimi, Diego Romano Perinelli, Giulia Bonacucina

**Affiliations:** 1 Chemistry Interdisciplinary Project (ChIP), School of Pharmacy, University of Camerino, 62032 Camerino, Italy; eleonora.spinozzi@unicam.it (E.S.); marco.cespi@unicam.it (M.C.); marta.ferrati@unicam.it (M.F.); riccardo.petrelli@unicam.it (R.P.); filippo.maggi@unicam.it (F.M.); giulia.bonacucina@unicam.it (G.B.); 2 School of Biosciences and Veterinary Medicine, University of Camerino, 62032 Camerino, Italy; junbiao.wang@gmail.com (J.W.); sundaysegun.alimi@studenti.unicam.it (S.S.A.)

**Keywords:** natural extracts, alkylamide, dynamic light scattering, nanoemulsion-based hydrogel, microemulsions, rheology, permeation study

## Abstract

**Background/Objectives**: Natural products are gaining increasing importance due to the large variety of biological activities exerted by their constituents. Among these, the products deriving from *Acmella oleracea* (L.) R.K. Jansen can be exploited for their local anaesthetic, myorelaxant, anti-inflammatory/analgesic, and antifungal properties. In this regard, there is a need to develop novel formulations for the topical delivery of *A. oleracea*-derived extracts to widen their use in the pharmaceutical and cosmetic fields. **Methods**: Nanoformulations, i.e., nanoemulsions (NEs) and microemulsions (MEs), were investigated as a strategy to encapsulate an extract from *A. oleracea* at the nanoscale level in water and then incorporated into xanthan gum-based hydrogels. **Results**: Only NEs provided a physically stable formulation, while the precipitation of solid hydrophobic components from the extract was observed during ME preparation under all tested conditions despite the use of ethyl oleate as an oily co-solvent. The optimized NE-based hydrogel remained physically stable over six months, as confirmed by rheological measurements and polarized optical microscope observation, without a phase separation phenomenon. Therefore, NEs resulted more suitable nanodispersed systems than MEs for the encapsulation of *A. oleracea* extract, which contains a large amount of hydrophobic constituents that are solid at room temperature. Furthermore, the sustained spilanthol release across an artificial membrane (Franz cell apparatus) and the cytotoxic profile on HaCaT cell line support its potential topical application. **Conclusions**: The outcomes of this study provided valuable insights into the formulation of *A. oleracea* extract, broadening its fields of applicability, including topical administration.

## 1. Introduction

Botanicals are becoming increasingly important in the pharmaceutical, nutraceutical, cosmetic, and agrochemical fields thanks to the large variety of secondary metabolites encompassed in their phytocomplexes, able to exert different functional activities [1,2,3]. However, the real-world application of natural substances is often hindered by their unfavourable physicochemical properties, with one of the main challenges being the development of effective delivery systems. In fact, these compounds are generally lipophilic and chemically unstable, making them susceptible to degradation [4,5]. Moreover, their low water solubility and unsuitable molecular size result in poor absorption and limited physiological availability [6,7]. To overcome these issues, various formulation strategies have been explored to enhance solubility, protect against degradation, reduce potential toxicity, improve bioavailability, and, where possible, boost the biological activity of the formulated products [8,9,10]. One of the most common formulating approaches is nanoencapsulation, through which natural compounds are loaded (or adsorbed) inside carriers of nanometric dimensions able to enhance the stability, physicochemical properties, and bioactivity of these products [11,12].

Among the various nanosystems, microemulsions (MEs) and nanoemulsions (NEs) are among the simplest to formulate and manage, and they can be produced at a relatively low cost [13]. These systems are nanoscale colloidal dispersions composed of two immiscible liquid phases (an oil phase and an aqueous phase) stabilized by surfactants [14]. Although their names include the prefixes “micro-” and “nano-”, both MEs and NEs feature a dispersed internal phase consisting of nanometric droplets [15,16]. The main differences between them lie in their quantitative composition and system free energy. Specifically, NEs typically have a lower surfactant-to-oil ratio (SOR), generally ranging from 1 to 2, compared to MEs, which usually require an SOR greater than 2 [17]. On the other hand, MEs are thermodynamically stable and form spontaneously, while NE formation needs external input to overcome high energy barriers [15,18]. These nanocarriers have been largely employed in the formulation of natural hydrophobic products such as essential oils and plant extracts [19,20].

*Acmella oleracea* (L.) R. K Jansen (Asteraceae) is a plant native to South America that recently garnered increasing research interest [21]. Indeed, it is characterized by a rich phytocomplex constituted by a wide range of secondary metabolites such as *N*-alkylamides, flavonoids, tannins, and saponins [22]. Among these, the most interesting group of compounds are the *N*-alkylamides, mainly represented by spilanthol, to which the plant’s main biological properties can be ascribed [23,24,25]. These compounds possess pharmacological effects that make them exploitable for a topical application as local anaesthetic [26], anti-inflammatory/analgesic [27,28,29,30], and antifungal agents [31]. Moreover, *A. oleracea* extract has been recently proposed as a potent natural botox alternative, thanks to its anti-wrinkle effect generated by its reversible myorelaxant activity and its promotion of collagen production [32,33]. For the abovementioned reasons, *A. oleracea*-derived products can be promising candidates as functional ingredients for the formulation of cosmetic, nutraceutical, and pharmaceutical products. Despite *A. oleracea* extracts having been chemically characterized and formulated [28,34,35] as emulsions or creams [36], no studies have aimed at their encapsulation into NEs and MEs and their formulation into a hydrogel. Specifically, only NEs and MEs prepared using *A. oleracea* essential oil are reported in the literature [37]. The encapsulation into nanodisperse aqueous systems could improve the absorption of the active components across the skin and the formulation of a hydrogel can ameliorate the contact feeling and texture of the formulation with respect to emulsions and creams. Therefore, the present study aimed to investigate the feasibility of preparing NEs and MEs from an *N*-alkylamide-rich extract obtained from *A. oleracea* and to prepare a semisolid hydrogel formulation based on these nanocarriers to enlarge the field of applicability of *A. oleracea*-derived products.

## 2. Materials and Methods

### 2.1. Materials

Ethyl oleate (Crodamol™ EO) was provided by Croda (Snaith, UK). Polysorbate 80 (Tween^®^ 80), activated charcoal, Celite^®^, acetonitrile, and methanol (HPLC-grade) were purchased by Merck KGaA (Darmstadt, Germany). Glycerol, ethanol 96% *v*/*v*, and n-hexane were acquired from Carlo Erba (Cornaredo, Italy). Xanthan gum was purchased from ACEF Spa (Fiorenzuola D’arda, Italy).

### 2.2. Plant Materials

Flowering aerial parts of *A. oleracea* var. oleracea were sourced from Dr. Ettore Drenaggi’s plantation. The seeds were sown between May and June 2021 in the midsection of the Musone riverbed, located in Castelfidardo, Italy (43°27′16″ N; 13°31′52″ E). After harvesting, the aerial parts were dried at 40 °C for 72 h and subsequently ground using a grinder (Albrigi, model E0585, Stallavena, Verona, Italy) equipped with a 1.5 mm pore-size sieve [38].

### 2.3. Preparation of A. oleracea n-Hexane Extract

The flowering aerial parts of *A. oleracea* (100 g) were extracted with n-hexane (1:10 *w*/*v*) using an ultrasound bath extractor (Analogic ultrasonic bath, Model AU-220, ARGOLAB, Carpi, Italy) at 30 °C for 3 h. The extract was filtered, and the solvent was removed under reduced pressure with a rotary evaporator (Buchi Rotavapor R-200, Büchi Labortechnik AG, Flawil, Switzerland) at 40 °C, yielding 11.5 g of extract (corresponding to 11.5% *w*/*w* of the dry weight). Chlorophyll removal was then carried out by dissolving the crude extract in 50 mL of n-hexane, adding activated charcoal (5.7 g, equivalent to 50% *w*/*w* of the dry extract), and stirring the mixture at 30 °C for 1 h. The mixture was subsequently filtered through Celite^®^ and evaporated to dryness, obtaining 9.0 g of extract. Further purification was performed by winterization: 5 g of the extract was dissolved again in n-hexane (1:10 *w*/*v*) and stored at 4 °C for 24 h to promote the precipitation of insoluble materials, which were then removed by filtration. This procedure was repeated twice. The final purified extract (PE) was dried (4.5 g yield) and stored at 4 °C until further use [38].

### 2.4. HPLC-DAD-MS Analysis of N-Alkylamides in A. oleracea Extract

The identification and quantification of *N*-alkylamides in *A. oleracea* n-hexane extract were performed using the method previously published [38,39]. The analysis was performed with an HPLC instrument Agilent 1100 series (Agilent Technologies, Santa Clara, CA, USA), constituting a photodiode array detector (DAD), an autosampler, a binary solvent pump, and an ion-trap mass spectrometer (with an electrospray ion source) from LC/MSD Trap SL Agilent Technologies, controlled by LCMSD (Agilent, v.6.2) and ChemStation (Agilent, v.01.03) software. The column was a Luna C18 column (4.6 × 150 mm, i.d., particle size 5 μm) from Phenomenex (Chesire, UK), operating at 35 °C. The analytical conditions were those already published [38,39]. The quantification of *N*-alkylamides was performed employing a spilanthol calibration curve. The latter was constructed by injecting spilanthol solution in methanol at different concentrations (1700, 850, 170, 85, 17, 8.5, and 1.7 mg/L) and by plotting them against the obtained analyte peak areas. The n-hexane extract was solubilized in acetonitrile at 1000 mg/L and the solution was filtered through a 0.2 μm syringeless filter before the analysis.

### 2.5. Nanoemulsion (NE) Formulation

The NEs were prepared using a high-energy homogenization method (French cell press, AMINCO, New York, NY, USA) starting from the corresponding coarse emulsions, according to the procedure reported in [40]. The oil phase was formed by the *A. oleracea* extract alone or previously solubilized at room temperature into ethyl oleate. The selected surfactant was polysorbate 80. Different samples were prepared by changing the amount of the components at the selected extract to ethyl oleate weight ratio (1:0, 1:1, and 1:2), and surfactant-to-oil-phase weight ratio (0.125, 0.25, and 0.375) to optimize the formulation process (Table 1).

### 2.6. Microemulsion (ME) Development

MEs were prepared upon mixing the components (oil phase, surfactant, and aqueous phase) according to the procedure described in [41]. The oily phase was constituted by *A. oleracea* extract and ethyl oleate at the weight ratio 1:1 (total oil phase 1% *w*/*w* or 0.5% *w*/*w*). Polysorbate 80 was employed as a surfactant (10, 13, and 16%, *w*/*w*). The aqueous phase was ultrapure water alone or in the presence of an alcoholic mixture (35%, *w*/*w*) formed by glycerol and ethanol at a weight ratio of 6:1. The composition (%) of all samples prepared is reported in Table 2.

### 2.7. Characterization of Micro- (MEs) and Nanoemulsions (NEs)

All the prepared NEs and MEs were firstly evaluated by visual inspection and using polarizing optical microscopy (MT9000, Meiji Techno Co., Ltd., Saitama, Japan, equipped with a 3-megapixel CMOS camera, Invenio 3S, DeltaPix, Smørum, Denmark). Droplet size distributions at 25 °C were collected through dynamic light scattering (DLS) using a Zetasizer nanoS (Malvern Instrument, Malvern, UK) equipped with a backscattered light detector working at 173°. DLS analyses were performed after sample preparation (T0) for all NEs and MEs. For NE_3, NE_6, and NE_7, DLS analyses were also conducted at different time points (T30 days, T9 days, and T180 days) to assess their physical long-term stability. During the stability period, samples were stored at room temperature and protected from light.

### 2.8. Preparation of A. oleracea Extract NE-Based Hydrogels

The hydrogels were prepared by dispersing the adequate amount of xanthan gum in the *A. oleracea* extract NE to obtain a final concentration of the polymer of 1.5, 2, and 2.5% *w*/*w*. Methyl p-hydroxy benzoate was employed as a preservative at the concentration of 0.2% *w*/*w*. Control hydrogels were prepared at the same concentrations in preserved ultrapure water. After the preparation, the hydrogels were stored at 4 °C in tight-closed vials protected from light.

### 2.9. Rheological Characterization of A. oleracea Extract NE-Based Hydrogels

Rheological properties of the *A. oleracea* extract NE-based hydrogels were determined according to the stress sweep and frequency sweep test. The stress sweep test was carried out at a frequency of 1 Hz at 25 °C in a range of stress between 0.1 and 100 Pa. The frequency sweep test was carried out at a stress of 1 Pa at 25 °C in a range of frequency between 0.1 and 10 Hz. Both tests were carried out the day after the preparation of the hydrogels. Samples prepared in water were also analysed as controls. Stress sweep tests were also repeated after one month, two months, and three months from the preparation to assess the stability of the formulations in terms of consistency.

### 2.10. Permeability Studies Using Vertical Diffusion Cells

The release and permeation of spilanthol from *A. oleracea* NE-based hydrogel, prepared using 2% *w*/*w* of xanthan gum, was evaluated at 32 ± 1 °C using vertical diffusion cells (Franz cell-type apparatus, Teledyne Hanson Research, Chatsworth, CA, USA) and employing a regenerated cellulose dyalisis membrane (cut-off 6–8 kDa, SpectraPor, Fischer Scientific, Pittsburgh, PA, USA). For each experiment, the receptor cell was filled with 7 mL of a water/propyleneglycol mixture at a 70:30 ratio and the donor cell was loaded with 300 µL of the hydrogel or the NE. The diffusion area of the membrane was 1.583 cm^2^. The system was continuously stirred at 600 rpm for six h and 0.5 mL of samples was withdrawn at each time point (0, 30, 60, 90, 150, 210, 300, and 360 min) and replaced with fresh solvent. Five replicates were carried out (n = 5). The amount of released spilanthol was monitored using a UV-vis spectrometer (UV-1800, Shimadzu, Kyoto, Japan) monitoring a wavelength of 228 nm and constructing a calibration curve (y = 0.1148x + 0.0343). The results are expressed as the spilanthol amount permeated (µg/cm^2^) ± SD and the stationary flux (J, µg/cm^2^/h) ± SD of five independent replicates.

### 2.11. Cell Cytotoxicity Study on HaCaT Cell Line

Immortalized human keratinocyte cells (HaCaTs) were obtained from the ISZLER cell culture bank (Brescia, Italy) and maintained in Dulbecco’s Modified Eagle Medium (DMEM, CORNING, Mediatech) supplemented with 10% foetal bovine serum (FBS, Gibco, Life Technologies, Carlsbad, CA, USA,) and 1% penicillin–streptomycin (Gibco, Life Technologies, Carlsbad, CA, USA). Cells were incubated at 37 °C in a humidified atmosphere containing 5% CO_2_. For the viability assay, HaCaT cells were seeded at a density of 3000 cells per well in 96-well plates containing complete medium. After 24 h, the medium was replaced with fresh medium containing various concentrations of *A. oleracea* extract (0.5–150 µg/mL), *A. oleracea*-loaded NE (0.5–49.6 mg/mL), or NE alone as a control (5–125 mg/mL). Cell viability was assessed after 72 h using the MTT assay [3-(4,5-dimethylthiazol-2-yl)-2,5-diphenyl-2H-tetrazolium bromide; Sigma Aldrich, St. Louis, MO, USA], which measures the reduction in MTT to insoluble formazan by mitochondrial enzymes. Formazan crystals were solubilized in DMSO, and the absorbance was measured at 540 nm using the FLUOstar Omega Plate Reader (BMG Labtech, Ortenberg, Germany). Each concentration was tested in six replicates, and the experiments were independently repeated three times. The data were analysed using GraphPad Prism 10 software. Half-maximal inhibitory concentration (IC_50_) values were determined by fitting the concentration–effect data to a sigmoid-Emax model through nonlinear regression, applying a weighting factor equal to the reciprocal of the square of the predicted effect. The results are expressed as the mean of IC_50_ values and at the 95% interval of confidence.

## 3. Results

### 3.1. HPLC-MS-DAD Analysis of N-Alkylamides in A. oleracea Extract

The HPLC-MS-DAD analysis led to the identification and quantification of six *N*-alkylamides as reported in Table 3. The six *N*-alkylamides were identified by comparing the MS spectra with those reported in the literature as (2Z)-*N*-isobutyl-2-nonene-6,8-diynamide (A1), (2E)-*N*-isobutyl-2-undecene-8,10-diynamide (A2), (2E,6Z,8E)-*N*-isobutyl-2,6,8-decatrienamide or spilanthol (A3), (2E,7Z)-*N*-isobutyl-2,7-decadienamide (A4), (2E)-*N*-(2-methylbutyl)-2-undecene-8,10-diynamide (A5), and (2E,6Z,8E)-*N*-(2-methylbutyl)-2,6,8-decatrienamide (A6). The main *N*-alkylamide found in both extracts was spilanthol, accounting for 12.8 and 8.2 g/100 g of RE and PE, respectively. This slight difference in spilanthol content could be ascribed to the winterization process that could lead to a partial loss of the compound during the precipitation of the insoluble materials. Moreover, the concentration of spilanthol in the RE extract was lower (12.8 vs. 24.8 g/100 g of dry extract) than that found in the work of Boukouvala et al. [39]. This result could be ascribed to the choice of performing a depigmentation process to facilitate the formulation step.

### 3.2. Formulation Study and Stability of Nanoemulsions (NEs)

**Figure 1:**
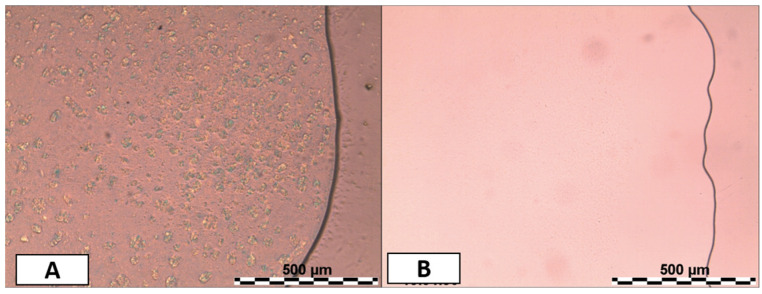
Polarized optical microscope images of *A. oleracea* extract (**A**) and the mixture of *A. oleracea* and ethyl oleate at the weight ratio 1:1 (**B**) (magnification 10×).

Initially, a formulation study was carried out to investigate the optimal amount of *A. oleracea* extract and surfactant to prepare NEs. As an extract containing a mixture of liquid and solid components at room temperature (Figure 1A), its solubilization in a suitable diluent oil was required. As the oil phase, ethyl oleate was selected, thanks to its good solubilization capacity and well-established use in the formulation of NEs and MEs [41]. The minimum weight ratio of *A. oleracea* extract and ethyl oleate required to obtain a stable solution at room temperature—without the precipitation of any solid compounds—was determined to be 1:1 (Figure 1B).

The need to dilute the extract in an oil phase (as ethyl oleate, at the weight ratio 1:1) to achieve a nanodispersed formulation was confirmed as follows. NEs without ethyl oleate (NE_1) and with different *A. oleracea* extract to ethyl oleate ratios (1:0.5; 1:1) (NE_2 and NE_3) were prepared using a fixed concentration of polysorbate 80 at 0.5% *w*/*w*. Indeed, the presence of solid particles was observed in the NE without ethyl oleate (NE_1) and in the NE prepared at the extract-to-ethyl oleate ratio of 1:0.5 (NE_2). An isotropic system was obtained when 1:1 and 1:2 extract-to-ethyl oleate ratios were employed (NE_3 and NE_6). Therefore, it was confirmed that the weight ratio 1:1 between *A. oleracea* extract and ethyl oleate was suitable for NE preparation. Then, the effect of different amounts of surfactant at the same extract/ethyl oleate (1:1) weight ratio was evaluated and three NEs were prepared containing polysorbate 80 at the concentrations of 0.25, 0.50, and 0.75% *w*/*w* (NE_4, NE_3, and NE_5, respectively). These concentrations of polysorbate 80 correspond to a surfactant-to-oil phase ratio of 0.125, 0.25, and 0.375, respectively. All these three NEs (NE_3, NE_4, and NE_5) showed a monomodal droplet size distribution in the nanometric range and a mean droplet size in the range of 150–220 nm (Figure S1). From these size distributions, the Z-average, representing an average droplet size value, and the polydispersity index (PDI), representing an adimensional parameter (ranging from 0 to 1) indicating the width of the droplet size distribution, were calculated. The Z-average and PDI values of all prepared NEs are reported in Table 4.

The concentration of surfactant in relation to the amount of the oil phase exerted a marked effect both on Z-average and PDI values of the produced NEs. Indeed, NE_4, having the lowest concentration of polysorbate 80 (0.25% *w*/*w*), showed Z-average and PDI values larger than those prepared using higher concentrations (0.5% *w*/*w* and 0.75% *w*/*w*). No marked differences between NE_3 and NE_5 were observed in terms of particle size and distribution, suggesting that a polysorbate to oil phase ratio ≥ 0.25 is enough to prepare NEs with a Z-average below 150 nm and a PDI value below 0.2.

**Figure 2:**
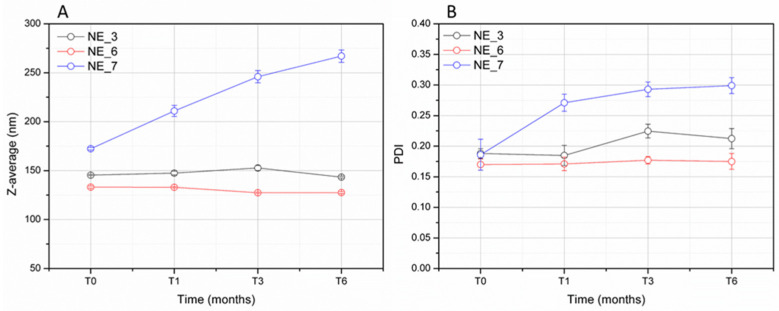
Variation in Z-average (**A**) and PDI (**B**) of NE_3, containing 0.5% *w*/*w* of polysorbate 80, NE_6 and NE_7 (surfactant-to-oil ratio of 0.25) over time up to 6 months of storage at room temperature.

The physical stability of the NE prepared at a surfactant-to-oil ratio of 0.25 (NE_3, NE_6, and NE_7), which is the minimum tested ratio ensuring a Z-average below 150 nm and a PDI value below 0.2, was assessed in terms of Z-average and PDI values during storage at room temperature for 6 months. The measured Z-average values of NE_3 and NE_6 remained stable over time at around 130–150 nm for all the period of observation. As regards the PDI, only a slight increase at around 0.21–0.22 was observed after 3 months of storage for NE_3, while the measured PDI of NE_6 did not change up to 6 months remaining at around 0.17–0.18 (Figure 2). From these results, it could be argued that ethyl oleate is not only able to effectively solubilize the solid compounds of the *A. oleracea* extract but it can also have a positive effect on particle size distribution, since the NE containing more ethyl oleate maintained PDI values below 0.2. NE_7, despite having a weight ratio of 1:1 between the extract and ethyl oleate as NE_3 (prepared with a double amount, 4% *w*/*w*, of extract and ethyl oleate), displayed a gradual increase in Z-average (from ~170 to 270 nm) and PDI values (from ~0.19 to 0.30) over time, suggesting a low grade of physical stability with respect to the other tested NEs, although no phase separation occurred according to the visual inspection. NE_3, containing 0.5% *w*/*w* of polysorbate 80 and the minimum amount of ethyl oleate required for the solubilization and the NE physical stability (1:1 extract to ethyl oleate weight ratio), was selected for further investigations.

### 3.3. Formulation Study and Stability of Microemulsions (MEs)

**Figure 3:**
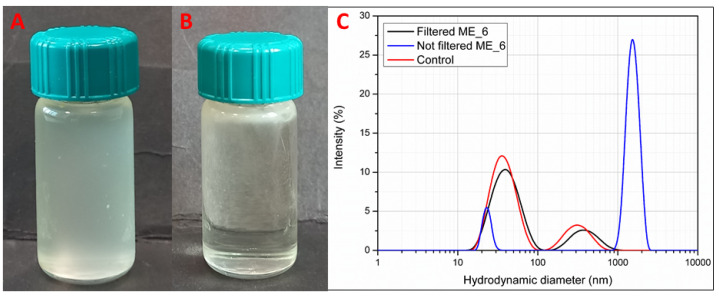
The physical appearance of ME_6 after preparation (**A**) and after one week (**B**) stored at room temperature. DLS traces for ME_6 after one week from the preparation before and after filtration in comparison to the ME control (0.5% ethyl oleate without *A. oleracea* extract) and aqueous phase control (polysorbate 80, alcohol mixture, and water) (**C**).

The formulation of MEs (M_1–M_12) was carried out by varying the percentage of polysorbate 80 (10, 13, and 16%, *w*/*w*) and the percentage of the extract (1 and 0.5%, *w*/*w*) previously solubilized in ethyl oleate at the weight ratio 1:1 as for the preparation of the NEs. The effect of the presence of co-surfactants in the aqueous phase was also evaluated. To this extent, MEs were prepared in an alcoholic mixture (35% *w*/*w* of the final composition) (M_1–M_6) or in water (M_7–M_10). All the formulations did not provide transparent samples (Figure 3A) due to the presence of dispersed solid materials, undergoing layering on the top of the samples over time (Figure 3B). Thus, none of the prepared formulations can be considered true MEs. Solid materials are also visible in all samples under optical and polarized microscope observation (Figure S2A). These solid materials were formed in the samples during the preparation of MEs probably due to the phase separation of some components of the extracts. This occurred despite the extract being previously solubilized in ethyl oleate at a ratio of 1 to 1 as for the formulation of NEs. Notably, no droplets of micrometric size were detected in these samples under optical microscope observation, suggesting that the lack of transparency under visual observation is not related to the formation of an emulsified system (Figure S2A). Subsequently, after the removal of the solid particles by syringe filtration (membrane pore size 0.45 µm), all systems appeared isotropic, like the MEs (Figure S2B). Moreover, the collected layered solid materials resemble the morphological aspect of the crude extract showing solid birefringent particles (Figure S2C). Figure 3C shows DLS traces for ME_6 (as a reference sample) before and after filtration in comparison to the control ME (the same composition of the ME without the extract). ME_6 displayed a multimodal profile according to intensity (%) size distribution, having a size population above 1000 nm before filtration, which could be ascribed to the presence of solid particles in dispersion, in addition to the other two populations in the nanometric range. After the filtration process was performed to remove the solid particles, the DLS trace was similar to those previously reported for MEs prepared with polysorbate 80, with the first population centred at around 50 nm and the second population at around 500 nm [41]. Therefore, according to the visual and microscopy observations and DLS results, MEs, different to NEs, seem to be unsuitable formulations for *A. oleracea* extract, since isotropic liquid nanodispersions do not form due to the presence of solid components.

### 3.4. Rheological Characterization of the A. oleracea NE-Based Hydrogels

**Figure 4:**
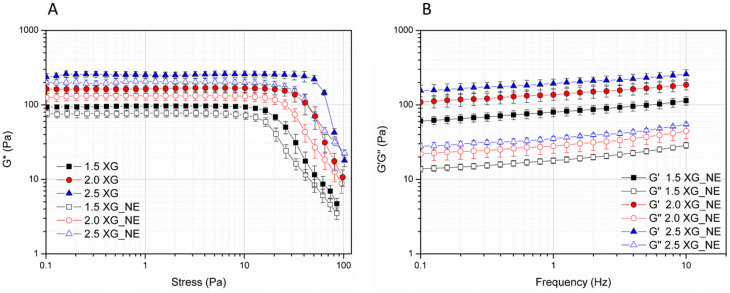
Stress sweep test at 25 °C (**A**) and frequency sweep test at 25 °C (**B**) for xanthan gum-based hydrogels at different concentrations (1.5, 2.0, and 2.5% *w*/*w*) prepared in water (1.5 XG, 2.0 XG, and 2.5 XG) and in NE_3 (1.5 XG_NE, 2.0 XG NE, and 2.5 XG_NE).

Figure 4A shows the variation in the G′ modulus for the NE-based hydrogels at three concentrations of xanthan gum (1.5%, 2%, and 2.5% *w*/*w*, respectively) prepared using NE_3 as a medium in comparison to those prepared in water. NE_3 was selected based on previous stability results: unlike NE_6 and NE_7, NE_3 contains the minimum amount of ethyl oleate required to obtain a stable nanoemulsion. The values of G′ inside the linear viscoelastic region (LVR) can be considered as a measure of the consistency of the hydrogels [42]. Indeed, LVR represents the plateau-like part of stress sweep plots in which the rheological characteristics of the samples are constant and independent of the applied stress. In this region, G′ values were dependent on polymer concentration and in the range of 70–300 Pa. It can be noted that the nanoemulsion-based hydrogels displayed a consistency only slightly lower than those prepared in water. A further investigation of the rheological properties of the hydrogels can be performed through a frequency sweep test, by investigating the variation in the rheological storage modulus G′ and loss modulus (G″) over frequency. As for the hydrogel prepared in water (Figure S3), for all nanoemulsion-based hydrogels, the values of G′ are higher than G″ values in all ranges of the frequencies analysed (1–10 Hz) (Figure 4B). This means that the solid or elastic component of the oscillatory response to the applied stress is larger than the liquid or viscous one. Therefore, it seems that nanoemulsion-based hydrogels behave as solid-like viscoelastic materials, able to store the energy inside their internal three-dimensional ordered structure [43]. Consequently, both moduli show little dependency on the frequency and have similar values in hydrogels prepared in water or NE as medium. For a better comparison and to underline the effect of NE as a medium on the viscoelastic properties of the hydrogels, G′ and G″ curves from the frequency tested were fitted using the “power law” equation modified with the yield parameter.

G′ = Y′ + K′ ωn′(1)

G″ = Y″ + K″ ωn″(2)

where ω is the applied frequency, n′ and n″ are the apparent viscoelastic exponents, K′ and K″ are the consistency indexes, and Y’ and Y″ are the yield values representing the values of the moduli at a frequency approaching zero [44].

The variation in G′ and G″ moduli are expected to be fitted through the power law model since this equation describes their relaxation dependence on the applied frequencies [45,46].

The three calculated parameters (Y, K, and n) are reported in Table S1. The calculated n′ and n″ values were much lower than 1, indicating the larger prevalence of the elastic behaviour for all hydrogels. Moreover, the n′ and n″ values for the NE-based hydrogels were slightly higher than those of the corresponding hydrogels prepared in water at the same percentage of xanthan gum, suggesting a slightly more pronounced influence of the frequency on both rheological moduli. All hydrogels had a yield value much higher than 0 as for real jellified systems in which the polymer forms a 3D internal network and K values reflect the effect of the xanthan gum concentration and the medium on the consistency of the systems.

The physical stability over time up to 90 days of the NE-based hydrogels prepared at three different concentrations of xanthan gum (1.5, 2, and 2.5% *w*/*w*) was assessed by visual and optical microscope observations and following the changes in the rheological parameter G′ measured at 1 Pa and 1 Hz by the stress sweep test. According to visual observation, no phase separation was macroscopically observed in all samples since they appeared as opaque cream-like systems over time (Figure S4). Moreover, according to optical microscopy observation, all samples did not show any detectable oily droplets in the micrometric range or the formation of birefringent solid particles, suggesting that for all the observation time, the mixture of the extract with ethyl oleate remained nanoemulsified inside the polymeric matrix of the hydrogel and the solid compounds of the extract did not crystallize (Figure S5). Rheological properties of the prepared NE-based hydrogels seemed also to be unaffected during the observation time, at least in terms of consistency, as resulted from the comparable G′ values measured at the different time points for all systems. Only for 2.5_XG_NE, a slight increase in the G′ value and large variability among the independent samples were observed (Figure S6).

### 3.5. Permeation Study

**Figure 5:**
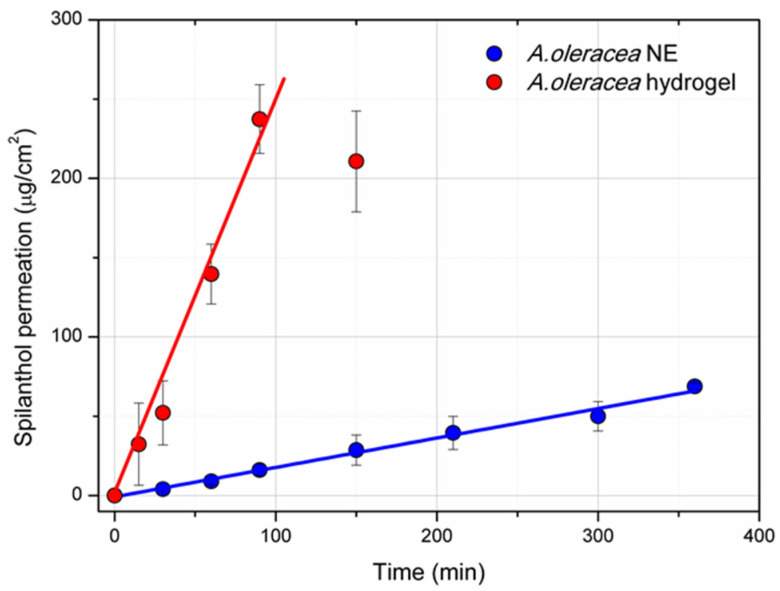
Permeation of spilanthol (µg/cm^2^) over time (min) from *A. oleracea* NE and *A. oleracea* hydrogel through cellulose dialysis membrane (cut-off 6–8 kDa) using Franz cell apparatus.

Spilanthol permeability across an artificial membrane was assessed on 2% *w*/*w* xanthan gum *A. oleracea* hydrogel (2.0 XG_NE) and *A. oleracea* NE served as the control (Figure 5). Both formulations showed a zero-order kinetics, meaning that a constant amount of the active compound can pass through the membrane. Indeed, the experimental data can be well fitted using a straight line (Adj.R^2^ = 0.989, slope = 0.294 for *A. oleracea* hydrogel; and Adj.R^2^ = 0.980, slope = 2.642 for *A. oleracea* NE), from which the stationary flux (J) across the membrane can be calculated. The calculated J was 17.64 µg/cm^2^/h and 158.52 µg/cm^2^/h for *A. oleracea* hydrogel and for *A. oleracea* NE, respectively. The total amount permeated was 68.7 µg/cm^2^ for *A. oleracea* hydrogel after 6 h and 237 µg/cm^2^ for *A. oleracea* NE after 90 min.

### 3.6. Cytotoxicity Study on HaCaT Cells

**Figure 6:**
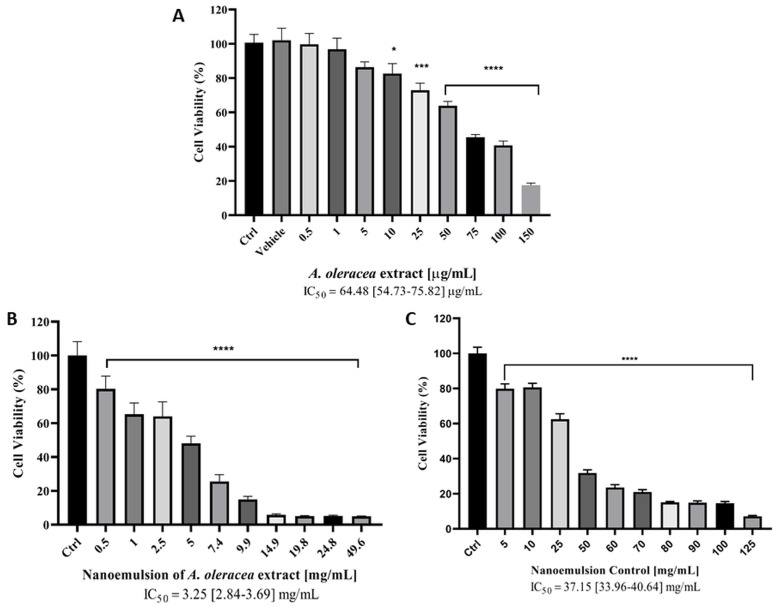
Cytotoxicity assay results on human keratinocyte (HaCaT) cell lines for *A. oleracea* extract (**A**) *A. oleracea* NE (**B**) and NE control without NE (**C**); cell viability is assessed using the MTT assay, and the results are expressed as the percentage of viable cells relative to the untreated control. Data represent the mean of three independent experiments, each performed with six technical replicates per treatment. SEM.* 0.01< *p* < 0.05, *** 0.0001< *p* < 0.005, **** *p* < 0.0001, one-way ANOVA followed by Dunnett’s multiple-comparisons test.

The cytotoxic effect of *A. oleracea* extract, *A. oleracea* NE (NE_3) and control NE was evaluated on an immortalized human keratinocyte cell line (HaCaT) as a model for the cutaneous application of *A. oleracea* formulations (Figure 6). Both *A. oleracea* extract and NE formulations showed a dose-dependent decrease in cell viability in the range of concentrations tested from which the IC_50_ values were calculated. Moderate cytotoxic activity was observed for the *A. oleracea* extract on HaCaT cells (IC_50_~64.48 µg/mL), which was comparable to that of spilanthol (>50 µg/mL) and supercritical fluid *A. oleracea* extract from the same cell line [47]. A higher IC_50_ value was calculated for *A. oleracea* NE (~3.25 mg/mL), reflecting the low content of the *A. oleracea* extract in the NE (1% *w*/*w*). Residual cytotoxicity was also observed for the control NE, which displayed an approximately ten times higher IC_50_ value (~37.15 mg/mL) with respect to that of *A. oleracea* NE, probably due to the presence of the surfactant polysorbate 80 in the NE formulation at the concentration of 0.5% *w*/*w*.

## 4. Discussions

NEs and MEs are nanoemulsified formulations that have been successfully employed for the vehiculation of essential oils in aqueous systems [20,37,41]. Particularly, these nano vehicles were effective in enhancing the bioactivity of the components of essential oil and enhancing their chemical–physical stability [48,49]. Despite the large number of studies regarding NEs and MEs for essential oils, these nanosystems have been not investigated in terms of loading other botanicals as natural extracts. Natural extracts have gained an increasing amount of interest in the field of formulation since they exhibit biological activity and promote the development of sustainable products [13,18,19,50]. For this reason, this research was devoted to the discovery and development of novel formulations suitable for different applications of these products.

*A. oleracea* is a natural source of bioactive secondary metabolites to which a broad spectrum of therapeutic properties can be attributed [51]. Only a few formulations based on plants from *Acmella* genus can be found in the literature such as a spray formulation based on the ethanolic extract of *A. oleracea* inflorescences for premature ejaculation [52], an emulsion-based hydrogel (emulgel) for the treatment of dermatitis [53] and an emulsion serum proposed as a natural anti-wrinkle product [36]. Moreover, some commercial products incorporate nanostructured lipid carriers loaded with *A. oleracea* extract, which are currently used for cosmetic applications [54]. As regards NEs and MEs based on *A. oleracea*, they have only been prepared starting from its essential oils obtained from dried flowers, stems, and leaves [37] but not starting from an extract of the same plant. In this study, both NEs and MEs of the *A. oleracea* extract, after the removal of chlorophyll and winterization processes, were investigated to determine the most suitable formulation for this natural product. NEs successfully encapsulated *A. oleracea* extract since they formed under all tested compositions. Specifically, the obtained droplet mean size for *A. oleracea* extract NEs measured by DLS was only slightly higher (120–218 nm for Z-average) than those for the *A. oleracea* essential oil NE (˂100 nm for Z-average), while the width of the droplet size distribution was similar (PDI values between 0.2 and 0.3) [37]. It should also be underlined that the total amount of the oil phase is lower for *A. oleracea* extract NEs (1-4% *w*/*w* of total oil phase) than for *A. oleracea* essential oil NEs (6–9% *w*/*w* of the total oil phase). On the contrary, the attempt to formulate *A. oleracea* extract into MEs did not provide isotropic nanodisperse systems since insoluble materials formed during their preparation, which layered on the top over time. This can be attributed to the precipitation of hydrophobic components of the extract, which are solid at room temperature, such as long-chain alkanes, alcohols, and fatty acids, together with possibly some *N*-alkylamides. Notably, the solubilization capacity of the ethyl oleate toward these compounds, as the oily phase at the tested oil-to-extract 1:1 ratio, remains effective in the case of the formulation using NEs, but not for the formulation using MEs. A possible explanation could be related to the lower particle size and different composition/flexibility of the interface in MEs than in NEs [55]. Moreover, the presence of a higher amount of surfactants in MEs configures these nanodispersions as “swollen micelle systems”, in which the oil phase is adsorbed onto the surfactant [15]. Hydrophobic solid components of the extracts may be not efficiently adsorbed onto the surfactant as hydrophobic liquids (such as oils) are. Therefore, they can form a precipitate in the aqueous environment of MEs.

One interesting application of *A. oleracea* extract is its use in a topical formulation in a range of concentrations 0.5–2% *w*/*w* as local anaesthetic, anti-inflammatory/analgesic, myorelaxant, and antifungal agents. Therefore, there is a need to develop semi-solid formulations suitable for its administration onto the skin. To this extent, the prepared NEs were incorporated into a xanthan gum-based hydrogel, which maintained its rheological properties after the loading with the extracts. Moreover, the NE-based hydrogels assured the physical stability of the extract (no oil droplets or formation of solid particles) during the observation time of three months. The suitability of the prepared formulation for topical applications onto the skin was preliminarily assessed through a permeation assay using an artificial membrane and evaluating the cytotoxic activity on keratinocytes (the HaCaT cell line). The ability of the *N*-alkylamide splilanthol to permeate across the skin has been already demonstrated [56] as well as the transdermal behaviour of its formulation as an emulsion [53,57]. However, the permeation properties of spilanthol when formulated into NE have never been investigated. The permeation assay conducted in this study demonstrates that the incorporation of the *A. oleracea* NE into a xanthan gum-based hydrogel can control the release of spilanthol from the formulation to the donor compartment according to zero-order kinetics, as resulted from the much lower calculated slope for the *A. oleracea* hydrogel with the respect to *A. oleracea* NE. The calculated permeation parameters such as J are in line with those obtained from other drugs using a similar apparatus and conditions [58,59]. As regards cytotoxicity studies, the *A. oleracea* NE showed a moderate toxicity (an IC_50_ value on the HaCaT cell line of ~3 mg/mL after 72h of exposure), which is promising for its application onto the skin. *A. oleracea* extract has an intrinsic cytotoxic effect on these types of cells (an IC_50_ value of ~64 µg/mL), which is much higher than that of the formulation (containing only 1% *w*/*w* of the extract) and comparable to that already reported in the literature (an IC_50_ value of >50 µg/mL) [46].

## 5. Conclusions

The present work focused on the feasibility of preparing nanodisperse systems such as MEs and NEs from *A. oleracea* extract and their incorporation into a hydrogel-based formulation. In detail, NEs were suitable vehicles for the incorporation of the *A. oleracea* extract in a liquid aqueous dispersion after its solubilization in ethyl oleate (1 to 1 weight ratio), demonstrating nanometric droplet size distribution and physical stability for up to six months. On the contrary, ME formulations encapsulating *A. oleracea* extract could not be achieved, at least under the investigated conditions, since the formation of insoluble materials was observed, probably due to the precipitation of hydrophobic solid components from the extract. The obtained results suggest that NEs could be more effective than MEs in the formulation of natural plant extracts that have a large content of solid chemical compounds. A stable hydrogel formulation based on NEs, showing a sustained permeation and moderate cytotoxicity, was prepared with the aim of developing a novel semisolid formulation for *A. oleracea* and to enlarge the fields of applicability of this natural product.

## Figures and Tables

**Table 1:** Composition (% *w*/*w*) of prepared nanoemulsions (NEs) based on *A. oleracea* extract [38].

Sample	Extract (%)	Ethyl Oleate (%)	Polysorbate 80 (%)	H_2_O (%)
NE_1	1	-	0.50	98.50
NE_2	1	0.5	0.50	98.00
NE_3	1	1	0.50	97.50
NE_4	1	1	0.25	97.75
NE_5	1	1	0.75	97.25
NE_6	1	2	0.75	96.25
NE_7	2	2	1.00	95.00

**Table 2:** Composition (% *w*/*w*) of prepared microemulsions (MEs) based on *A. oleracea* extract [38].

Sample	*A. oleracea* Extract (%)	Ethyl Oleate (%)	Polysorbate 80 (%)	Alcoholic Mixture (%)	H_2_O (%)
ME_1	1	1	10	35	53
ME_2	1	1	13	35	50
ME_3	1	1	16	35	47
ME_4	0.5	0.5	10	35	54
ME_5	0.5	0.5	13	35	51
ME_6	0.5	0.5	16	35	48
ME_7	1	1	10	0	88
ME_8	1	1	13	0	85
ME_9	1	1	16	0	82
ME_10	0.5	0.5	10	0	89
ME_11	0.5	0.5	13	0	86
ME_12	0.5	0.5	16	0	83

**Table 3:** *N*-alkylamides identified and quantified in *A. oleracea* n-hexane extracts (RE and PE).

	*N*-Alkylamide	[MH]^+^ (m/z)	[M + NH_4_]^+^ (m/z)	[M + Na]^+^ (m/z)	RE ^a^		PE ^e^	
					Concentration (g/100 g RE) ^b^ ± SD ^c^	RSD % ^d^	Concentration (g/100 g PE) ± SD	RSD %
A1	(2*Z*-*N*-isobutyl-2-nonene-6,8-diynamide	204.7	222.7	-	0.1 ± 0.0	6.3	0.1 ± 0.0	9.9
A2	(2*E*)-*N*-isobutyl-2-undecene-8,10-diynamide	232.6	-	254.6	tr ^f^	5.7	tr	12.8
A3	(2*E*,6*Z*,8*E*)-*N*-isobutyl-2,6,8-decatrienamide (spilanthol)	222.5	-	244.5	12.8 ± 0.2	1.7	8.2 ± 0.0	0.5
A4	(2*E*,7*Z*)-*N*-isobutyl-2,7-decadienamide	224.6	-	246.6	0.1 ± 0.0	1.8	0.1 ± 0.0	20.3
A5	(2*E*)-*N*-(2-methylbutyl)-2-undecene-8,10-diynamide	268.6	-	268.6				
A6	(2*E*,6*Z*,8*E*)-*N*-(2-methylbutyl)-2,6,8-decatrienamide	236.6	-	258.6	0.9 ± 0.0	2.9	0.6 ± 0.0	0.2
	Total	-	-	-	13.9 ± 0.2	1.8	8.9 ± 0.0	0.4

^a^ RE, dry raw extract; ^b^ average concentration in 100 g of RE; ^c^ SD, standard deviation; ^d^ RSD %, relative SD; ^e^ PE, dry purified extract; ^f^ tr, traces (concentration ≤ 0.05).

**Table 4:** Z-average and polydispersity index (PDI) values obtained after dynamic light scattering (DLS) analysis of nanoemulsions (NE_3–NE_7) after their preparation measured at 25 °C.

	Z-Average (nm)	PDI
NE_3	148.7 ± 0.6	0.178 ± 0.015
NE_4	216.5 ± 1.6	0.274 ± 0.002
NE_5	121.1 ± 0.7	0.198 ± 0.014
NE_6	133.2 ± 1.1	0.170 ± 0.009
NE_7	172.4 ± 1.2	0.186 ± 0.025

## Data Availability

The original contributions presented in this study are included in the article/Supplementary Material. Further inquiries can be directed to the corresponding author.

## Supplementary Materials

The following supporting information can be downloaded at: https://www.mdpi.com/article/10.3390/pharmaceutics17050625/s1, Figure S1: Particle size distribution (intensity %) of prepared NEs (NE_3, NE_4, and NE_5) at 25 °C. Figure S2: Optical polarized microscope image of microemulsions after preparation (A) after syringe filtration (regenerate cellulose, pores 0.45 µm) (B) and solid materials layered on top of microemulsions over time (C). Figure S3: Frequency sweep test at 25 °C for xanthan gum-based hydrogels prepared in water (1.5 XG, 2.0 XG and 2.5 XG). Figure S4: An image of the 2.0 XG_NE sample after 90 days from the preparation. Figure S5: Optical polarized microscope images of the nanoemulsion-based hydrogels at different xanthan gum concentrations (1.5% *w*/*w*, 2.0% *w*/*w* and 2.5% *w*/*w*). Figure S6: Variation in G′ value at 1 Pa over time for xanthan gum-based hydrogels at different concentrations (1.5, 2.0, and 2.5% *w*/*w*) prepared in NE_3 (1.5_XG_NE, 2.0_XG_NE and 2.5_XG_NE). The reported values are the mean ± standard deviation of three independent measurements. Table S1 Rheological parameters (Y, K, and n) as determined by the power law with yield equation applied for all the prepared hydrogels.
