# Supplementary material for: Hydrogels Powered by Nanoemulsion Technology for the Topical Delivery of Acmella oleracea Extract
Source: Pharmaceutics. 2025 May 8;17(5):625. doi: 10.3390/pharmaceutics17050625 (PMC12115319; doi:10.3390/pharmaceutics17050625)
Supplement: Supplementary file 1 [file pharmaceutics-17-00625-s001.zip › pharmaceutics-3582763-supplementary.pdf]

Supplementary Materials

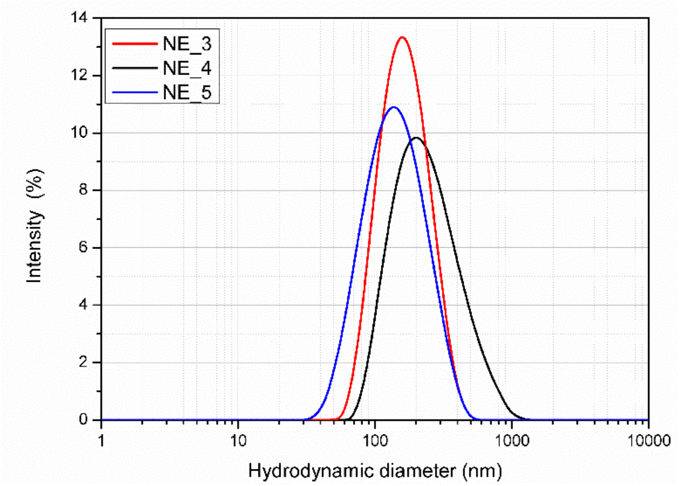

Figure S1. Particle size distribution (Intensity %) of prepared NEs (NE_3, NE_4, and NE_5) at 25°C.

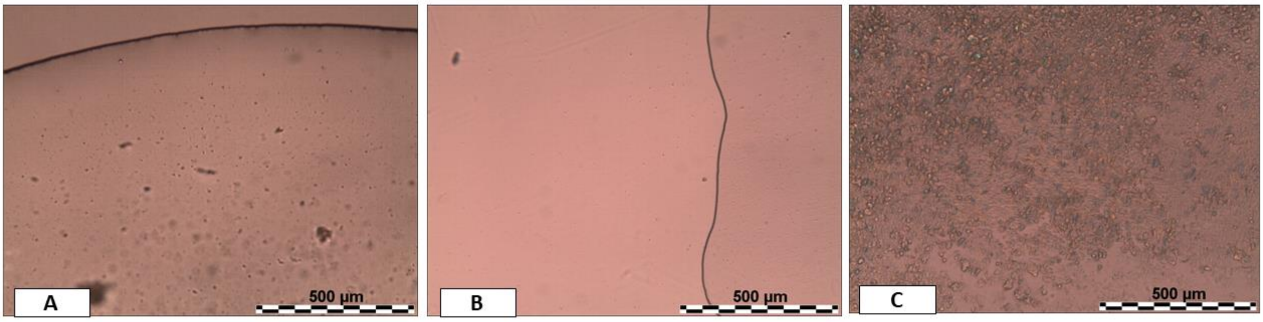

Figure S2 Optical polarised microscope of microemulsions after preparation (A), after syringe filtration (regenerate cellulose, pores 0.45 μm) (B) and solid materials layered on the top of microemulsions over time (C).

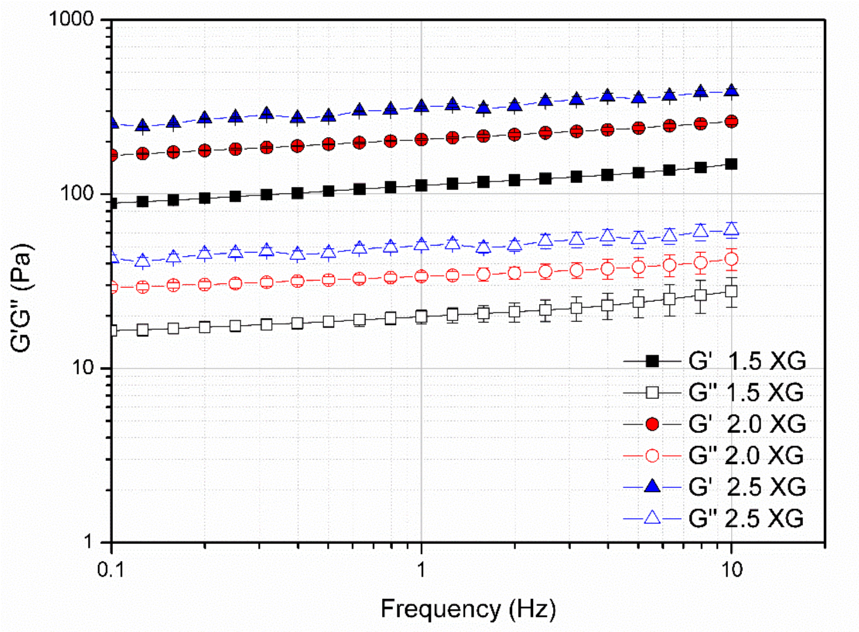

Figure S3 Frequency sweep test at 25 °C for the xanthan gum-based hydrogels prepared in water (1.5 XG, 2.0 XG and 2.5 XG)

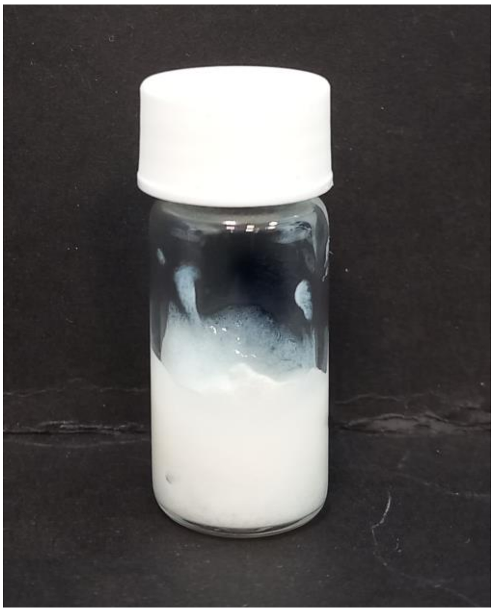

Figure S4 Image of the 2.0 XG_NE sample after 90 days from the preparation

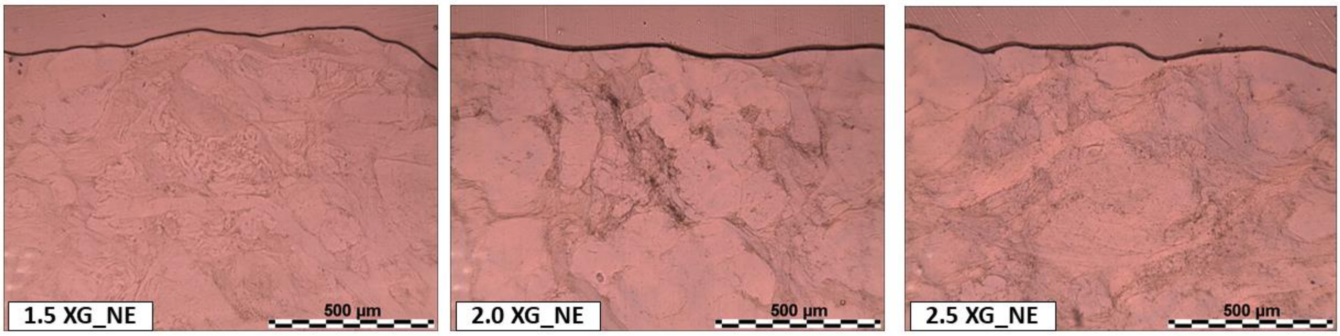

Figure S5 Optical polarised microscope images for the nanoemulsion-based hydrogels at different xanthan gum concentrations (1.5 % w/w, 2.0% w/w and 2.5% w/w).

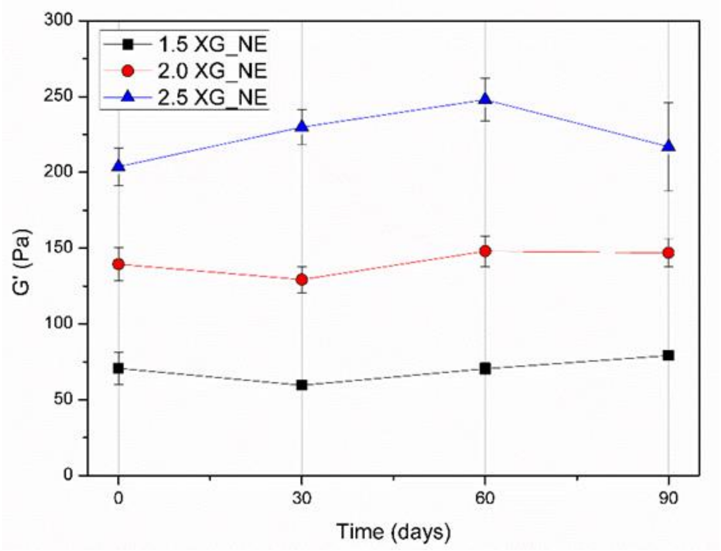

Figure S6. Variation of G' value at 1 Pa over time for the xanthan gum-based hydrogels at different concentrations (1.5, 2.0, and 2.5% w/w) prepared in NE_3 (1.5_XG_NE, 2.0_XG_NE and 2.5_XG_NE). The reported values are the mean ± standard deviation of three independent measurements.

Table S1 Rheological parameters (Y, K and n) as determined by the power law with yield equation applied on the all the prepared hydrogels.

Samples	Elastic modulus *G'* $G' = Y + K' \omega^{n'}$				Viscous modulus *G''* $G'' = Y + K'' \omega^{n''}$			
	Y	K	n	R^2	Y	K	n	R^2
1.5% XG	36.67±5.55	77.14±6.06	0.161±0.009	0.992±0.004	14.77±1.06	6.60±3.44	0.386±0.070	0.989±0.007
2.0% XG	66.35±11.0	121.6±3.8	0.149±0.006	0.999±0.000	23.36±2.82	9.21±1.55	0.360±0.080	0.997±0.004
2.5% XG	89.34±5.46	232.1±10.8	0.124±0.005	0.975±0.003	30.22±1.92	16.17±3.67	0.289±0.008	0.967±0.009
1.5% XG_NE	38.28±0.47	41.07±5.31	0.256±0.024	0.998±0.001	11.86±1-40	5.91±0.13	0.446±0.018	0.999±0.001
2.0% XG_NE	66.56±0.33	71.61±9.32	0.224±0.053	0.997±0.002	19.48±3.79	8.54±2.18	0.462±0.017	0.998±0.002
2.5% XG_NE	95.54±1.54	97.84±14.8	0.227±0.078	0.994±0.006	24.80±3.78	10.27±1.56	0.458±0.044	0.995±0.004
